# Violaxanthin and Zeaxanthin May Replace Lutein at the L1 Site of LHCII, Conserving the Interactions with Surrounding Chlorophylls and the Capability of Triplet–Triplet Energy Transfer

**DOI:** 10.3390/ijms23094812

**Published:** 2022-04-27

**Authors:** Donatella Carbonera, Alessandro Agostini, Marco Bortolus, Luca Dall’Osto, Roberto Bassi

**Affiliations:** 1 Department of Chemical Sciences, University of Padova, via Marzolo 1, 35131 Padova, Italy; alessandro.agostini@umbr.cas.cz (A.A.); marco.bortolus@unipd.it (M.B.); 2 Biology Centre, Czech Academy of Sciences, Institute of Plant Molecular Biology, Branišovská 1160/31, 370 05 České Budějovice, Czech Republic; 3 Department of Biotechnology, University of Verona, Strada Le Grazie, 37134 Verona, Italy; luca.dallosto@univr.it (L.D.); roberto.bassi@univr.it (R.B.)

**Keywords:** light-harvesting complex II, LHCII, carotenoid, TTET, TR-EPR, ODMR, triplet state

## Abstract

Carotenoids represent the first line of defence of photosystems against singlet oxygen (^1^O_2_) toxicity, because of their capacity to quench the chlorophyll triplet state (^3^Chl) through a physical mechanism based on the transfer of triplet excitation (triplet–triplet energy transfer, TTET). In previous works, we showed that the antenna LHCII is characterised by a robust photoprotective mechanism, able to adapt to the removal of individual chlorophylls while maintaining a remarkable capacity for ^3^Chl quenching. In this work, we investigated the effects on this quenching induced in LHCII by the replacement of the lutein bound at the L1 site with violaxanthin and zeaxanthin. We studied LHCII isolated from the *Arabidopsis thaliana* mutants *lut2*—in which lutein is replaced by violaxanthin—and *lut2 npq2*, in which all xanthophylls are replaced constitutively by zeaxanthin. We characterised the photophysics of these systems via optically detected magnetic resonance (ODMR) and time-resolved electron paramagnetic resonance (TR-EPR). We concluded that, in LHCII, lutein-binding sites have conserved characteristics, and ensure efficient TTET regardless of the identity of the carotenoid accommodated.

## 1. Introduction

**Figure 1:**
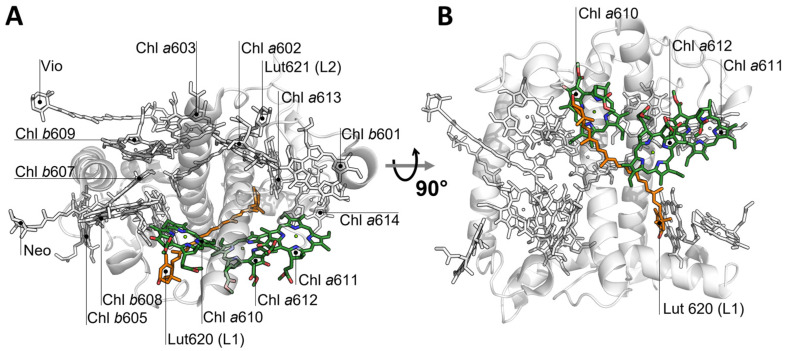
(**A**) Stromal view of LHCII (PDB ID: 1RWT, [2]), where the L1 site is highlighted. Green sticks: Chls *a*610, *a*611, *a*612; orange sticks: lutein at site L1 (Lut620); white sticks: other pigments; white cartoons: polypeptide chains; (**B**) 90° rotation perspective (membrane view).

Photosystem I (PSI) and photosystem II (PSII) are two large protein complexes comprising light-harvesting complexes (Lhc) that capture photons and transfer excitation energy to the reaction centres. Lhc proteins are organised around the photosynthetic reaction centres, forming supramolecular complexes embedded into the thylakoid membrane. Light-harvesting complex II (LHCII) is the major antenna of PSII, which is characterised by three transmembrane α-helices connected by hydrophilic loops, and a short amphiphilic helix exposed to the inner surface of the thylakoid membrane. Lutein (Lut) molecules are bound at binding sites L1 and L2; two additional sites located at the periphery of the complexes (N1 and V1) coordinate neoxanthin and violaxanthin/lutein, respectively [1]. Fourteen chlorophyll (Chl) molecules are coordinated to amino acid side-chains or to neighbouring Chl molecules, and are in tight connection with the carotenoid molecules [2]. Lut620 in L1 is close to Chls *a*610, *a*612, *a*613, and *a*614, while Lut621 in L2 is the nearest neighbour of Chls *a*602, *a*603, *b*607, and *b*609 (see Figure 1). During high light exposure, the amount of energy harvested exceeds the capacity for turnover of the reaction centres [3,4], and de-excitation mechanisms become operative to avoid the dangerous effects of triplet excited states centred on chlorophylls (^3^Chl) that, when reacting with oxygen, might produce singlet oxygen (^1^O_2_) and other reactive and harmful species [5]. Carotenoids (Car) may be considered the first line of defence of the photosystems against ^1^O_2_ toxicity, because of their capacity to quench ^3^Chl via triplet–triplet energy transfer (TTET) [6,7,8,9]. TTET is subsequently followed by thermal deactivation, without damage to the system. For TTET to occur, Chls and Cars must be in close proximity within the protein scaffold. In our recent work [9], the triplet states populated under illumination in monomeric LHCII were analysed via time-resolved electron paramagnetic resonance (TR-EPR) and optically detected magnetic resonance (ODMR) in A2 and A5 mutants, lacking Chls *a*612(*a*611) and Chl *a*603, respectively [10]. We found that, in the wild-type (WT) complex, only Lut620 was involved in triplet quenching in monomeric LHCII. In the A5 mutant, Lut620 retained this pivotal photoprotective role, while the A2 mutant was found to activate an alternative pathway for photoprotection, involving Lut621. These results indicate that LHCII is characterised by a robust photoprotective mechanism, able to adapt to the removal of specific chlorophylls while maintaining a remarkable degree of ^3^Chl quenching, by switching the role of the lutein molecules [11].

By studying recombinant LHCII samples with altered occupancy of xanthophyll sites, Formaggio et al. [12] found that the major contribution to protein stability is provided by interaction of the carotenoid bound to site L1 with Chl and protein moieties. The domains L2 and N1 weakly influence either the protein stability or the photoprotection; however, replacement of xanthophyll species bound to these structural domains modulates the fluorescence quantum yield of LHCII: binding of violaxanthin increases the fluorescence quantum yield with respect to Lut, while zeaxanthin decreases it. Recently, key interactions responsible for carotenoids’ binding to monomeric LHCII were analysed by combining molecular dynamics simulations and polarisable quantum mechanics/molecular mechanics calculations. The replacement of the native carotenoids with other xanthophylls did not produce marked changes in the interaction patterns in any binding pocket. This was particularly true for the L1 site, while somewhat higher flexibility was exhibited by the L2 site, since the latter seems to be more capable of tuning its hydrogen bond network based on the chemical properties of the xanthophyll [13]. Accordingly, Saccon et al. [14], based on the analysis of the spectroscopic changes due to differential binding of either lutein or violaxanthin, suggested that the LHCII protein scaffold evolved to be both flexible (allowing functional conformational switches) and robust (allowing redundancy in the nature of the xanthophylls bound). Unaltered probability to form Car triplet excited states in LHCII isolated from the *Arabidopsis thaliana* (*A. thaliana*) mutant *npq1lut2*—which cannot synthesise lutein, and cannot accumulate zeaxanthin—was also reported, and attributed to violaxanthin replacing the central lutein [15]. On the other hand, in *lut2* mutants, in which lutein is absent and substituted by violaxanthin, the trimerisation of LHCII complexes is prevented, and a higher level of photodamage in high light and/or low temperature is induced, suggesting that violaxanthin decreases the efficiency of ^3^Chl quenching [16]. Fractionation of thylakoid membranes from high-light-treated plants showed the presence of zeaxanthin in the monomeric Lhcb subunits of PSII (Lhcb4–6), but not in the LHCII complex. This presence was accompanied by a reduced yield of ^1^O_2_ production either from purified Lhc proteins or from photosynthetic supercomplexes containing these subunits, showing a direct involvement in the modulation of ^3^Chl [17].

In this work, we investigated the characteristics of the L1 site of LHCII by replacing lutein either with violaxanthin or zeaxanthin. To this aim, we studied LHCII isolated from *A. thaliana lut2*—in which lutein is replaced by violaxanthin—and *A. thaliana lut2 npq2*, in which all xanthophylls were replaced constitutively by zeaxanthin [18]. Thus, we were able to investigate the effects produced in LHCII by substitution not only with violaxanthin, as previously reported [15], but also with zeaxanthin. In particular, we studied the effects on the spectroscopic properties of the photoexcited triplet states and the efficiency of the TTET underlying one of the photoprotection mechanisms played by carotenoids, exploiting the unique sensitivity of ODMR and TR-EPR for this kind of process [9,19,20,21,22,23]. From the data, we concluded that the lutein-binding site L1 in LHCII can accommodate all three xanthophylls, maintaining the same pathway of TTET regardless of the identity of the carotenoid, without requiring rearrangements of the nearby chlorophyll molecules.

## 2. Results

### 2.1. Time-Resolved Electron Paramagnetic Resonance (TR-EPR) 

**Figure 2:**
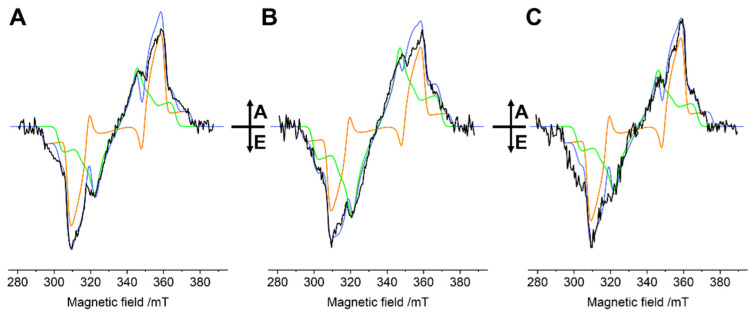
TR-EPR spectra (black) of (**A**) WT, (**B**) *lut2*, and (**C**) *lut2 npq2* LHCII variants taken 150 ns after the laser flash: All of the spectra were collected at 120 K and rescaled to the same concentration. Assignment according to Ref. [20]. Simulations of the TR-EPR spectra (light blue) obtained from the sum of the simulations of ^3^Car (orange) and ^3^Chl (green), optimised on the low-field half. The simulated ^3^Car spectra were calculated using the following parameters: D = −41.0 mT for WT and *lut2*, −41.4 mT for *lut2 npq2*; E = −4.0 mT; (P_x_:P_y_:P_z_) = (0.41:0.21:0.38; isotropic linewidth lw = 2.5 mT. The simulated ^3^Chl *a* spectra were calculated using the following parameters: D = −32 mT for WT, −35 mT for *lut2*, and −33 mT for *lut2 npq2*; E = −3.0 mT; (P_x_:P_y_:P_z_) = (0.36:0.44:0.20); isotropic linewidth lw = 3.5 mT. The simulations have a ^3^Car/^3^Chl ratio of 1.11 for WT, 0.67 for *lut2*, and 1.08 for *lut2 npq2* (note that the ratio is not indicative of the relative triplet amount, since polarisation strongly affects the TR-EPR signal intensity).

The 120 K TR-EPR spectra of the WT and *lut2*/*lut2 npq2* mutants of LHCII, detected 150 ns after the laser flash, show signals that can be assigned to ^3^Car and ^3^Chl, these triplet species having largely overlapping spectra (Figure 2). The spectra are very similar to those previously published for the same kind of LHCII preparations in monomeric form [11].

At a 150 ns delay after the laser flash, the Chl to Car TTET process was completed [24], and the polarisation of the ^3^Car could be considered to have been inherited from the ^3^Chl during TTET, because the polarisation of the detected triplet states had not yet started to change under the effect of the anisotropic relaxation of the triplet sublevels [9]. At the same time, “unquenched” ^3^Chls, which are not able to transfer their triplet state to Cars, were detected previously in LHCII [20], and were also present in all of our samples, although to a different extent; *lut2* presented a slightly higher amount of unquenched ^3^Chls, while WT and the *lut2 npq2* mutant presented a similar contribution (see Figure 2 for reconstruction of the spectra with ^3^Car and ^3^Chl contributions).

The presence of ^3^Chl *a* is commonly observed in isolated light-harvesting complexes [20,22,25,26,27], especially at low temperatures. It is worth noting that the polarisation pattern of the Car triplet state is the same in the three samples (i.e., *EEA/EAA*; see Figure 2), and only slight differences are present in the positions of the main turning points, indicating small differences in the zero-field splitting (ZFS) parameters. 

### 2.2. Optically Detected Magnetic Resonance (ODMR) of ^3^Car

As seen in the previous section, the different xanthophyll occupancy of the sites leads to only small changes in the triplet state EPR spectra, in terms of both relative ^3^Chl/^3^Car contribution and the ZFS parameters of the ^3^Car components. To draw a clearer picture of the impact of the carotenoid substitutions, and find correlations between the magnetic and optical properties of the chromophores involved in triplet formation, we performed ODMR experiments, in both absorption and fluorescence detection modes.

**Figure 3:**
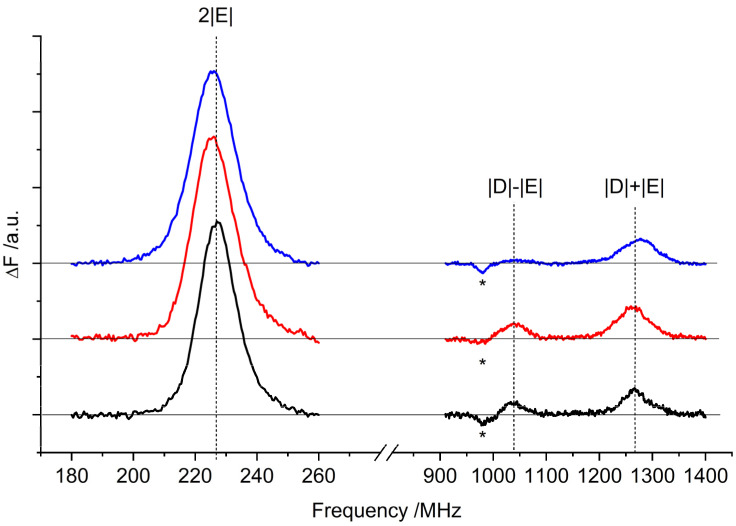
^3^Car FDMR spectra of WT (black), *lut2* (red), and *lut2 npq2* (blue) LHCII variants detected at 680 nm. Amplitude modulation frequency: 333 Hz; temperature: 1.8 K. The FDMR spectra were vertically translated for a better comparison. Note that the negative component to the left-hand side of the |D| − |E| transition (indicated by an asterisk) is assigned to a chlorophyll triplet (see below).

The ^3^Car fluorescence-detected magnetic resonance (FDMR) spectra obtained by monitoring the fluorescence changes of the samples while sweeping the microwave frequency are shown in Figure 3. The possibility of detecting non-fluorescent species—such as carotenoids—through the changes in Chl emissions is a well-known FDMR effect due to the energy transfer processes connecting the two pigments [19,28]. The ZFS parameters of the ^3^Car were derived from the transitions, and are reported in Table 1. The |D| − |E| transitions overlap with signals originating from ^3^Chl (appearing as negative peaks in the corresponding figure).

**Figure 4:**
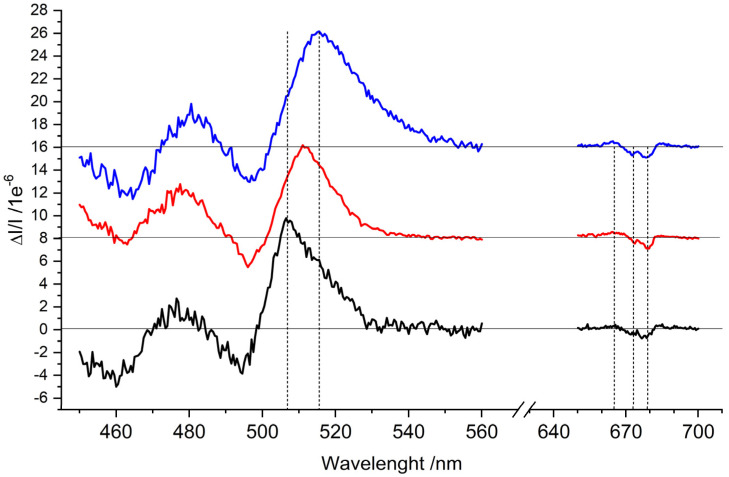
^3^Car T–S spectra of the WT (black), *lut2* (red), and *lut2 npq2* (blue) LHCII variants. Resonance frequency of about 225 MHz, amplitude modulation of 333 Hz, time constant of 3 s, and temperature of 1.8 K. The spectra of the *lut2* mutant (red) and the *lut2 npq2* mutant (blue) were vertically translated for a better comparison. The vertical dashed lines highlight the main transitions.

The ^3^Car triplet-minus-singlet (T–S) spectra of the samples obtained by fixing the microwave frequency at the maximum of the 2|E| transition observed in the FDMR spectra (Figure 3), and monitoring the change in the absorption, are reported in Figure 4. The spectra of the three samples show positive bands corresponding to triplet–triplet (T–T) absorption, along with negative bands corresponding to singlet–singlet absorption bleaching of Car species (Figure 4). The sample containing only zeaxanthin (*lut2 npq2*) shows a 10 nm redshift compared to the WT in the main positive band (516 vs. 506 nm), while the sample containing violaxanthin (*lut2*) shows a minor shift (511 vs. 506 nm). In the T–S spectra, structured bands are also present in the Q_y_ absorption region of Chl *a* (around 675 nm). These kinds of bands, often present in the T–S spectra of ^3^Car in photosynthetic systems, were previously assigned to the interaction with Chls located in the proximity of the Car carrying the triplet state at the L1 site, which “feel” the change in the electronic state of the Lut620 going from singlet to triplet [29,30,31,32]. It is clear that both violaxanthin and zeaxanthin are able to maintain their location at the site without significantly altering the interactions with the chlorophylls, since the same negative bands at 673 and 679 nm, as well as the positive one at 663 nm, were observed in all of the samples, with only small differences in their relative intensity. Moreover, the relative orientation of the Chl–Car pigments involved in TTET does not undergo changes after the introduction of violaxanthin or zeaxanthin, as proven by the polarisation pattern of the ^3^Car observed in the TR-EPR spectra in Figure 2, which was the same for all of the samples. This is consistent with a retained capability of quenching the Chl triplet states in the two mutants, which strongly depends on the relative orientation of the Chl and Car moieties [23].

### 2.3. Optically Detected Magnetic Resonance of ^3^Chl

**Figure 5:**
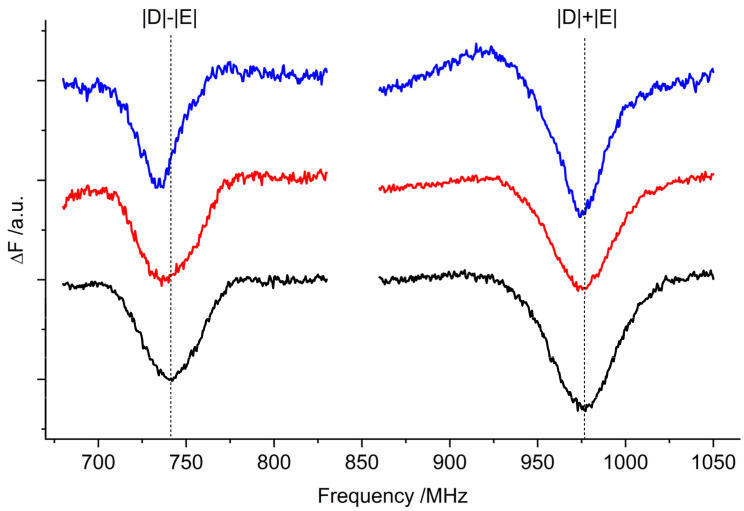
^3^Chl *a* FDMR spectra of the WT (black), *lut2* (red), and *lut2 npq2* (blue) LHCII variants detected at 690 nm. Amplitude modulation frequency: 33 Hz; temperature: 1.8 K.

As revealed by the TR-EPR spectra of LHCII, the quenching of the triplet state by Lut is not complete, and unquenched ^3^Chls are present. FDMR signals for all three samples were detected, varying the wavelength of detection between 680 and 690 nm, in the microwave field regions where the |D| − |E| and |D| + |E| transitions of ^3^Chl states were expected (600–1100 MHz) (Figure 5) [19,27]. As commonly observed, the 2|E| transition of ^3^Chl was too weak to be detected, while the |D| − |E| and |D| + |E| transitions were detected with comparable intensity. The intensity of the observed triplet states detected at the maximum fluorescence (690 nm) was very similar for the three samples, and due to the scarce reproducibility of the matrix quality and illumination conditions, the small differences cannot be considered significant. The TR-EPR spectra showed that in *lut2* samples the unquenched triplet states were present in larger amounts. It must be noted, however, that different preparations show some variability in the ^3^Chl populations for all of the samples. In contrast to [16], we did not observe a reproducible decrease in the efficiency of ^3^Chl quenching by violaxanthin.

## 3. Discussion

Recently, the possibility of individually removing the Chls *a* that were considered the sites of triplet formation in LHCII [20] allowed us to show that, at very low a temperature (1.8 K), the only site of Chl triplet quenching in monomeric WT LHCII is Lut620—the lutein sitting at the L1 site [11]. In a previous work, Saccon et al. [15] showed that lutein-binding sites in LHCII have conserved characteristics. Moreover, comparing isolated monomers of LHCII complexes of WT or *npq1 lut2* plants, they found that the terminal emitting chlorophylls and the xanthophyll in the L1 pocket constitute the primary sites for energy quenching, regardless of the differential binding of either lutein or violaxanthin, suggesting redundancy in the nature of the bound xanthophyll [14].

**Figure 6:**
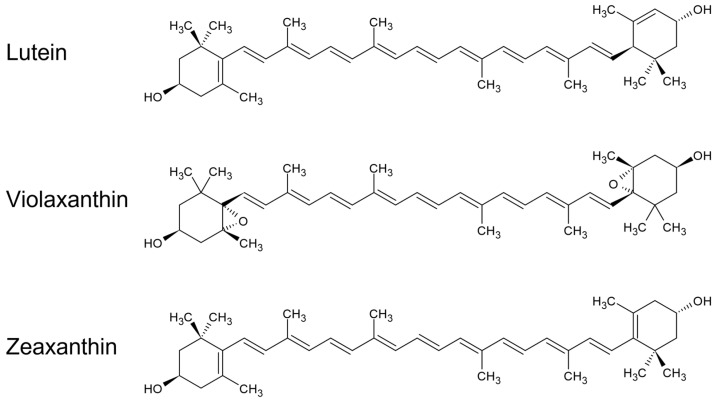
Molecular structure of lutein, violaxanthin, and zeaxanthin: In the *lut2* mutant, violaxanthin replaces lutein, whereas in the *lut2 npq2* mutant all xanthophylls are replaced constitutively by zeaxanthin.

In this work, we deeply investigated the changes in the spectroscopic properties of the carotenoid induced by its binding at the L1 site, with particular focus on the triplet state, and also extended the study to the mutant *lut2 npq2* containing only zeaxanthin. We found that in all of the samples ^3^Car were formed under illumination and at low temperatures, with comparable yield (as demonstrated by both the EPR and T–S experiments, Figure 2 and Figure 4, respectively). The ZFS of the three different carotenoids, measured precisely by FDMR, was almost identical (differences < 0.7%). This indicates that the electronic distribution in the triplet state, which strongly depends on conjugated chain length and molecular symmetry, is nearly the same in the three carotenoids. On the other hand, when observing the molecular structures reported in Figure 6, it is clear that the extension of the conjugated chain is expected to be different in all trans-carotenoids assuming planar configurations (violaxanthin having only 9 conjugated double bonds, lutein having 10, and zeaxanthin having 11). Accordingly, the absorption spectrum of the three carotenoids in organic solvent shows a redshift with the increasing number of conjugated carbon–carbon double bonds [33].

Thus, it seems that the binding to the L1 site (where carotenoid triplet states are formed [11,14,16]) flattens the differences in ZFS. 

Moreover, when observing the singlet–singlet (S–S) and T–T absorptions (appearing as negative and positive bands, respectively, in the T–S spectra reported in Figure 4), unexpectedly, violaxanthin did not exhibit a blueshift [33]. Instead, it showed a slight redshift (3–5 nm), confirming the room temperature absorption spectrum reported by Saccon et al. [15]. Zeaxanthin, in turn, exhibited a redshift compared to lutein, which was about 10 nm in the T–T main absorption band, consistent with the equivalent S–S absorption shift in dichloromethane [33]. A redshift in the major carotenoid signal in the T–S spectra upon zeaxanthin synthesis in high-light-treated plants was reported previously as being due to binding in monomeric Lhcb subunits of PSII (Lhcb4–6) and in the dimeric Lhca subunits of PSI, but not in the major trimeric LHCII complex. In each case, the zeaxanthin-dependent redshift was correlated with a reduced yield of ^1^O_2_ production from purified Lhc proteins or from photosynthetic supercomplexes containing these subunits [17].

Despite the differences in the maxima of the T–T absorption, the bands observed in the Chl Qy absorption region (between 650–700 nm) are well conserved for the three carotenoids. Since these bands are assigned to the changes in the absorption spectrum of the Chls, which “feel” the change in the electronic state of the carotenoid in which the triplet state becomes localised after light absorption, due to their spatial proximity, we may infer that the change in the structure of the carotenoid inserted at the L1 site does not alter the interactions with the nearby Chls—namely, Chl *a*610/(*a*611)/*a*612/*a*613. This result is consistent with recently published molecular dynamics simulations and polarisable quantum mechanics/molecular mechanics calculations performed on monomeric LHCII [13]. The calculations showed that 85% of the interaction of carotenoids with proteins and cofactors stems from van der Waals (vdW) forces, and that their major vdW interaction partners are just the surrounding Chl molecules; vdW stacking interactions, mostly involving xanthophyll’s conjugated linear chains, have much larger energy contributions than the energy ascribable to the hydrogen bonds of the side groups in the peripheral and flexible terminal rings. According to the calculations, the replacement of the native carotenoids with other xanthophylls (such as violaxanthin and astaxanthin) does not produce marked changes in the overall interaction patterns in the L1 binding pocket—neither to the vdW components nor to the hydrogen bond involving the lumenal –OH of L1 lutein and the –NH_2_ group of a conserved Gln residue. We could also extend these predictions to zeaxanthin, due to its similar structure—also characterised by –OH groups in the terminal rings. Compared to lutein, zeaxanthin has two terminal rings that are equivalent to those of lutein. It is likely that both rings may adopt in situ distortions similar to that observed for lutein. Thus, our observation of a conserved pattern in the Chl–xanthophyll interactions, as revealed by the T–S spectra in the Qy region, confirms the interchangeability of the xanthophyll at site L1 without requiring a rearrangement of the surrounding cofactors. Since the environmental flexibility is an important factor determining the excitation wavelength of the chromophore [34], we also expect that the dynamics of the L1 site are unchanged upon xanthophyll substitution. Results support the idea of the existence of a correlation between protein conformational flexibility and chlorophyll electronic transitions induced by light. In addition to the T–S spectra, the conserved polarisation pattern of the carotenoid triplet state of the TR-EPR spectra in the mutants also indicates that the Chl–xanthophyll pairs involved in TTET remain the same, with a conserved mutual geometry.

Our data show that carotenoid triplet formation is roughly the same for all of the complexes. The contribution of unquenched triplet states is on average not very different between the three samples, and is preparation-dependent to some extent.

It is worth noting that it has been reported previously that zeaxanthin may work actively in NPQ [35,36] by direct quenching of excited Chl singlet states [37], due to its specific capability to populate the CT state when a cofacial arrangement with nearby Chl is adopted [38,39]. Here, we show that at the L1 site, where a cofacial configuration with Chl612 is present, zeaxanthin does not preferentially quench Chl singlet states with respect to Lut or violaxanthin. Moreover, since our data refer to monomeric LHCII, we support the hypothesis that if CT states are formed at the L1 sites of LHCII [40], this certainly implies conformational changes compared to the monomeric configurations adopted in solution, where only the TTET mechanism is operative independently of the bound xanthophyll. Unquenched ^3^Chl was previously assigned to Chl *a*610 and Chl *a*602, with Chl *a*610 being dominant in WT LHCII [11]. We did not observe a significant increase in unquenched triplet states caused by substitution of native Lut with violaxanthin or zeaxanthin, meaning that the photoprotection path remained functional. The strong similarity in the ^3^Chl FDMR profiles points toward a conservation of the energetic landscape of the long absorbing Chls in the LHCII variants investigated, with Chl *a*610 being the main unquenched triplet trap of the Chl *a*610/*a*611/*a*612 red cluster facing the L1 site [11,41,42].

## 4. Materials and Methods

### 4.1. Sample Preparation

The wild type (WT), the double mutant *lut2 npq2*, and the *lut2* mutant of *A. thaliana* were grown according to the procedure previously described in [18]. Photosynthetic pigments extracted in methanol were separated and quantified by HPLC, as described in detail elsewhere [18]. Unstacked thylakoids were isolated from leaves that had been adapted to the dark for 2 h, as previously described [43]. Membranes were solubilised in 0.6% *w*/*v* α-DM (n-dodecyl-α-D-maltopyranoside) and 10 mM HEPES at pH 7.5, and then the solubilised samples were fractionated by ultracentrifugation in a 0.1–1.0 M sucrose gradient, with 0.06% *w*/*v* α-DM and 10 mM HEPES at pH 7.5, for 22 h at 280,000 g and 4 °C, as reported in [44]. Monomeric Lhcb and LHCII subunits were fractionated by flatbed isoelectric focusing at 4 °C [45]; green bands were harvested, and monomeric LHCs were further purified by sucrose gradient ultracentrifugation in 0.06% *w*/*v* α-DM. All complexes used in this work were in monomeric form for direct comparison, since trimerisation introduces different configurations in the L2 binding pocket [46]. Samples for the ODMR and TR-EPR experiments were diluted with degassed glycerol added to a final concentration of 60% (*v*/*v*) to obtain homogeneous and transparent matrices upon freezing. After the addition of glycerol, performed immediately before freezing to avoid sample degradation [47], the OD of the samples was about OD_670_ = 0.5 /mm (λ_exc_ = 670 nm). An optical pathway of 1 mm was used in all of the ODMR experiments. For TR-EPR experiments, 200 μL of each sample was added to quartz tubes (3 mm inner diameter).

### 4.2. ODMR Experiments

The principle of the ODMR technique has been previously reviewed in detail [19]. Fluorescence- (FDMR) and absorption-detected (triplet-minus-singlet, T–S) spectra were acquired in a home-built setup that has been previously described [48,49]. Briefly, a 250 W halogen lamp (Philips) was employed as a light source, filtered through either a 5 cm CuSO_4_ solution (FDMR) or a 10 cm water filter (T–S), and focused on the sample cell immersed in a helium bath cryostat. In FDMR experiments, the fluorescence was detected using a Si photodiode (OSI-Centronix) at 90° geometry with respect to the excitation light direction through appropriate bandpass filters (full width at half-maximum ∼10 nm), while in T–S experiments the transmittance was detected with a standard straight geometry through a monochromator (Jobin Yvon HR250), using the same detector as for FDMR measurements. Microwave-induced T–S spectra were obtained by fixing the microwaves at resonant frequency while sweeping the monochromator wavelength. The microwave resonator, where the sample cell is inserted, consists of a slow pitch helix. The microwaves, produced by a sweep oscillator (HP8559b) equipped with a plug-in (HP83522s) and amplified by a TWT amplifier (Sco-Nucletudes 10-46-30), were on/off amplitude-modulated for selective amplification, and the signals from the detector were demodulated and amplified using a lock-in amplifier (EG&G, mod 5210). The analogue output was connected to a computer-controlled analogue-to-digital converter. All relevant experimental parameters are reported in the figures.

### 4.3. TR-EPR Experiments

TR-EPR experiments were performed using a Bruker ELEXSYS E580 spectrometer, equipped with a dielectric cavity (Bruker ER 4117-DI5, TE_011_ mode), an Oxford CF935 liquid helium flow cryostat, and an Oxford ITC4 temperature controller. The microwave frequency was measured using a frequency counter (HP5342A). The temperature was controlled in a nitrogen flow, and all experiments were conducted at 120 K, disabling the magnetic field modulation and using pulsed-sample photoexcitation from a Nd:YAG pulsed laser (Quantel Brilliant) equipped with both second and third harmonic modules and an optical parametric oscillator (OPOTECH) (λ = 475 nm, pulse length = 5 ns, E/pulse ≅ 3.5 mJ, 10 Hz repetition time). The directly detected EPR signal was recorded with a LeCroy 9300 digital oscilloscope, triggered by the laser pulse. For every field position, 500 transient signals were averaged. The 2D field vs. time surfaces were corrected by subtracting both the EPR signal before the laser pulse and, to eliminate the laser background signal, the transients accumulated at off-resonance field positions. 

### 4.4. Triplet-State EPR Simulations

Simulations of the powder’s spin-polarised triplet spectra were performed using a program written in MATLAB^®^ (MathWorks, Natick, MA, USA), with the aid of the EasySpin routine (ver. 5.2.25) [50], based on the full diagonalisation of the triplet state spin Hamiltonian, including the Zeeman and electron–electron magnetic dipole interactions, considering a powder distribution of molecular orientations with respect to the magnetic field direction. Input parameters were the triplet-state sublevel populations, the zero-field splitting (ZFS) parameters, the linewidth, and the isotropic g value.

Calculations of the sublevel triplet-state populations of the acceptor (Car), starting from those of the donor (Chl), were performed using a home-written program in MATLAB^®^ software, following the formalism of Carbonera et al. [51], and utilising the X-ray coordinates of LHCII [2]. The program for the calculation of the triplet sublevel populations, previously described in great detail [20,52,53], lies in the limits of the high-field approximation and in the limits of a TTET process that is fast compared to the time evolution of the donor triplet spectrum (a process that requires hundreds of ns), but slow enough to allow spin alignment in the external magnetic field (a process that takes place in the picosecond time scale).

## 5. Conclusions

We were able to demonstrate that Lut620 may be replaced by both violaxanthin and zeaxanthin in ^3^Chl quenching, with comparable efficiency, by comparing the monomeric form of WT LHCII from *A. thaliana* with mutants *lut2* and *lut2 npq2* (replacing lutein with violaxanthin and zeaxanthin, respectively). Indeed, the substitution with violaxanthin and zeaxanthin did not significantly increase the unquenched ^3^Chl yield. Unquenched ^3^Chl was detected previously in WT LHCII by optical spectroscopy, even at room temperature, and represents only a few percent of the whole photoinduced triplet population in isolated LHCII [20,27,54]. 

We found that the changes in the structure of the carotenoid inserted at the L1 site do not alter the interactions with the nearby Chls—namely, Chl *a*610/(*a*611)/*a*612/*a*613—which are thought to be responsible for the main vdW interactions stabilising the binding of the carotenoid. The same pigment pair (Chl *a*612/Xan620) retains the photoprotective role as in the WT. 

In conclusion, our investigation provides further evidence that LHCII is characterised by a robust photoprotective mechanism, able to adapt to the substitution of various carotenoids because of a well-designed L1 site. The robustness of this photoprotective design is of pivotal importance, and is shared by the light-harvesting complex (LHC) superfamily [22]. 

It must be noted that the wild-type complement of xanthophylls, along with the light-dependent interconversion of xanthophyll pigments, is required to obtain functional flexibility for photosynthesis under rapidly fluctuating environmental conditions, i.e., a high efficiency of light harvesting in limiting light conditions and an efficient protection in high light. Zeaxanthin plays an important role in this balance. *lut2 npq2*, containing only zeaxanthin, has a small antenna system unable to perform state transitions, but an enhanced photosynthetic capacity in high light [16,17]. Thus, although the L1 site would be able to accommodate different xanthophylls with comparable performance, an active xanthophyll cycle confers an advantage over a constitutive expression of zeaxanthin (or violaxanthin), due to the specific role played by this carotenoid in many regulation processes [55,56,57].

## Figures and Tables

**Table 1:** ZFS parameters of ^3^Car determined from the FDMR and TR-EPR experiments.

	2|E| FDMR/MHz	|D| + |E| FDMR/MHz	|D| − |E| FDMR/MHz	|E| ^a^ FDMR/MHz	|D| ^b^ TR-EPR/MHz
**WT**	227 ± 1	1267 ± 1	1037 ± 0.1	113 ± 1	1152 ± 1
***lut2***	226 ± 1	1262 ± 1	1040 ± 0.1	113 ± 1	1151 ± 1
***lut2 npq2***	225 ± 1	1275 ± 1	1046 ± 0.1	112 ± 1	1160 ± 1

^a^ |E| parameters in mT: 40 mT for all samples. ^b^ |D| parameters in mT: WT = 411 mT; *lut2* = 410 mT; *lut2 npq2* = 414 mT.

## Data Availability

Data are available from the corresponding author upon request.
